# Complications of Benchekroun vesicostomy in a spina bifida patient: severe stenosis requiring permanent suprapubic cystostomy, recurrent vesical calculi and abdominal hernia containing ileocystoplasty - a case report

**DOI:** 10.1186/1757-1626-2-9371

**Published:** 2009-12-22

**Authors:** Subramanian Vaidyanathan, Bakul M Soni, Gurpreet Singh, Peter L Hughes, Paul Mansour, Tun Oo

**Affiliations:** 1 Spinal Injuries Unit, District General Hospital, Town Lane, Southport PR8 6PN, UK; 2 Department of Urology, District General Hospital, Southport PR8 6PN, UK; 3 Department of Radiology, District General Hospital, Southport PR8 6PN, UK; 4 Department of Cellular Pathology, District General Hospital, Southport PR8 6PN, UK

## Abstract

**Introduction:**

In female patients with neuropathic bladder, the urethra is closed permanently in order to avoid urine leak. Then Benchekroun hydraulic ileal valve is attached to urinary bladder, thus providing a continent stoma for performing intermittent catheterisations.

**Case presentation:**

We present a female patient with spina bifida who underwent Benchekroun continent vesicostomy in 1993. This patient developed severe stenosis of Benchekroun stoma and stones in urinary bladder. Dilatation of stoma and vesicolithotomy were carried out in 1995. Vesical calculi recurred; suprapubic cystolithotomy was performed in 1999. In March 2000, catheterisation of stoma was not possible and emergency suprapubic cystostomy was done. In April 2000, endoscopy was attempted through Benchekroun stoma. It was not possible to insert ureterorenoscope beyond two inches. The track was completely blocked. In November 2001, X-ray of abdomen showed several vesical calculi; suprapubic cystolithotomy was performed.

In March 2005, this patient developed pain in abdomen. X-ray of abdomen showed a large vesical calculus. In June 2005, suprapubic catheter was removed and a cystoscope was introduced in to the bladder. Then electrohydraulic lithotripsy was performed. In 2007, this patient was concerned about the increasing swelling in lower abdomen. Computed tomography of abdomen revealed midline, lower abdominal wall hernia, which contained several loops of small bowel and ileal cystoplasty. The large hernia was uncomfortable and tender on coughing, but did not cause obstructive bowel symptoms. Surgical repair of hernia was considered. But this patient would require alternative way of urinary diversion because the current location of suprapubic catheter would almost lead to infection of prosthetic material used in reconstruction of the anterior abdominal wall. After discussing risks of operative procedures with patient and her husband, it was decided not to proceed with surgery.

**Conclusion:**

This case is a poignant reminder to spinal cord physicians that novel surgical techniques should be viewed cautiously, and patients should be informed of potential complications of surgical procedures some of which could be irreversible.

## Introduction

Benchekroun hydraulic ileal valve is constructed by isolating a 14 cm long intestinal loop with its mesentery [1]. The isolated ileal segment is then folded inward on itself throughout its length. While performing Benchekroun continent vesicostomy in female patients with neuropathic bladder, the urethra is closed permanently in order to avoid urine leak. Then Benchekroun hydraulic ileal valve is attached to urinary bladder, thus providing a continent vesicostomy. The stoma of Benchekroun hydraulic ileal valve is sited in lower abdomen where it is readily accessible for self-catheterisation.

In female patients, urethral meatus may not be easily accessible for catheterisation. Some patients may develop patulous bladder neck and urethra; in these patients, urine leak between catheterisations may be a significant problem affecting quality of life. Therefore, Benchekroun hydraulic ileal valve appears to provide a viable solution for patients, who find difficulty in performing self-catheterisation through urethral meatus, or who leak urine between catheterisations despite taking anticholinergic drugs.

We present a female patient with spina bifida who underwent Benchekroun continent vesicostomy. This patient developed severe stenosis of stoma, marked dilatation of Benchekroun hydraulic valve, large abdominal ventral hernia containing ileocystoplasty, and recurrent vesical calculi. These complications severely compromised the quality of life for this patient. This case is a poignant reminder to spinal cord physicians that novel surgical techniques should be viewed cautiously, and patients should be informed of potential and sometimes, irreversible complications of surgical procedures.

## Case presentation

**Figure 1:**
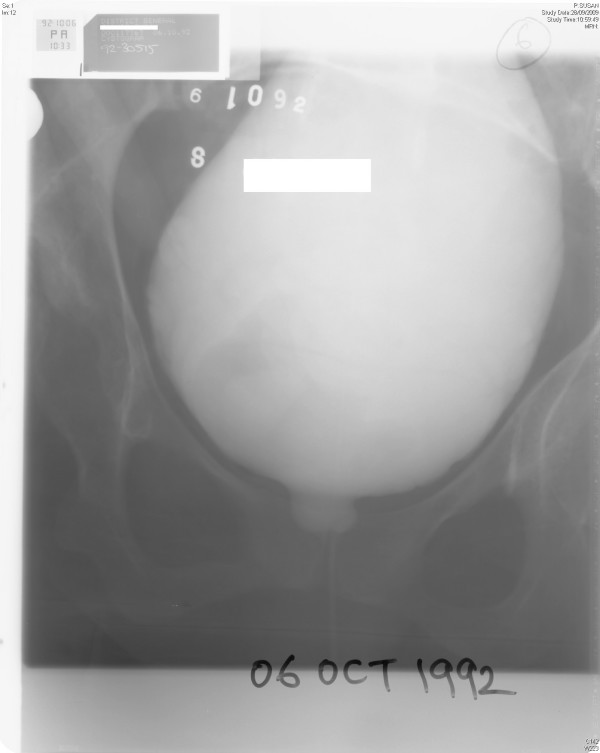
**Cystogram, done on 06 October 1992, revealed a large capacity bladder**. Bladder outline was smooth.

This British, Caucasian, female was born with spinal bifida in 1966. A by-pass for hydrocephalus was done in childhood. The ventriculo-peritoneal shunt was infected in 1988 and was changed to left side. Left ovary was removed when she was 16 years of age. Z plasty was performed for sacral sore on 05 January 1990. She was prescribed oxybutynin 5 mg nocte and she performed intermittent catheterisation four hourly. She was taking trimethoprim 100 mg nocte as prophylaxis for urine infection. Urodynamics study was done on 29 April 1992. This showed hypotonic bladder with absent detrusor reflex activity. Cystogram, done on 06 October 1992, revealed a bladder capacity of 800 ml. The bladder was big, smooth-walled. (Figure 1) There was no vesicoureteric reflux. There was no leak of urine per urethra. She was advised to perform self-catheterisations four per day. She was asked to limit intake to 1500 ml a day.

**Figure 2:**
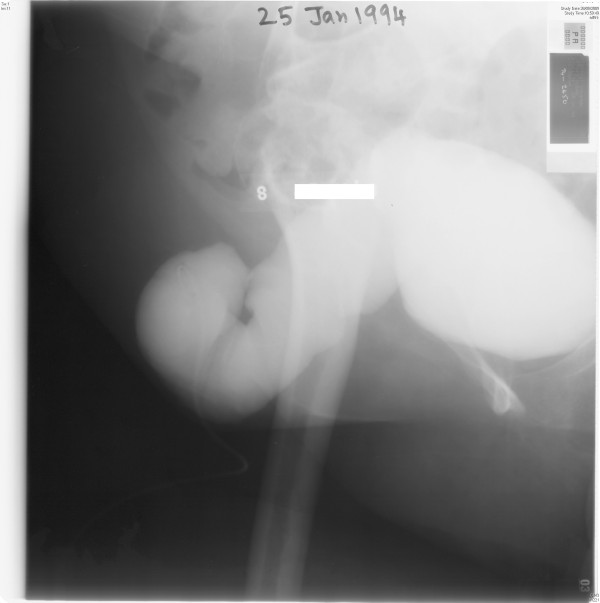
**Cystogram, performed on 25 January 1994, showed marked dilatation of ileal segment (Benchekroun valve)**.

Continent vesicostomy with Benchekroun hydraulic valve was performed on 17 June 1993. External urethral meatus was circumcised and urethra was dissected to bladder neck. A vertical midline suprapubic incision was made through the umbilicus. Urinary bladder was opened. Urethra was intussuscepted to bladder and excised. Bladder neck was closed in two layers. 14 cm ileum was isolated on mesentery; bowel continuity was restored. Mesenteric defect was closed. Ileum was intussuscepted with serosal surface outermost. The Benchekroun valve was fixed to apex of vesocostomy. The bladder was closed. Stoma was fixed to anterior abdominal wall in right iliac fossa extraperitoneally. A suprapubic cystostomy was kept emerging on left side of abdomen. On 12 July 1993, this patient was performing catheterisations through stoma; she was continent. On 24 November 1993, right peristomal herniation was noted. Cystogram, performed on 25 January 1994, showed dilatation of Benchekroun valve. (Figure 2) This patient was advised regular catheterisation.

Dilatation of Benchekroun stoma was performed on 21 July 1995. Open vesicolithotomy was carried out. The stoma had retracted to the back of abdominal wall. From there to the skin was a fibrous track, which had been kept open by catheterisations. Attempted dilatation with guide wire, Lister and open ended Clutton bougies allowed dilation of track but the cystoscope could not be passed along the track even over a guide wire. The track was dilated with Jacques catheters until a 14 Charriere catheter was passed in to urinary bladder. Suprapubic vesicostomy was made. Stones were delivered. A 20 Fr. 3-way catheter was left in bladder. Post-operative period was uneventful. Wound healed well. The Foley catheter was taken out and this patient started doing intermittent catheterisations.

**Figure 3:**
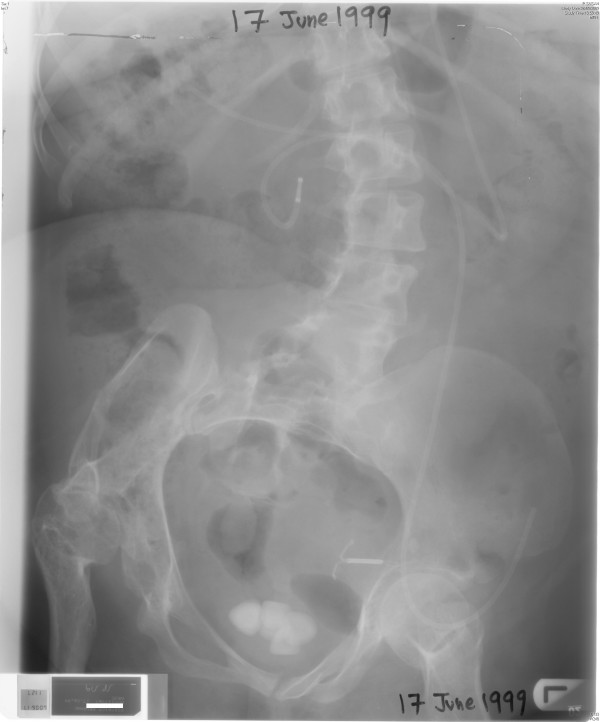
**X-ray of abdomen, taken on 17 June 1999, showed several vesical calculi**.

**Figure 4:**
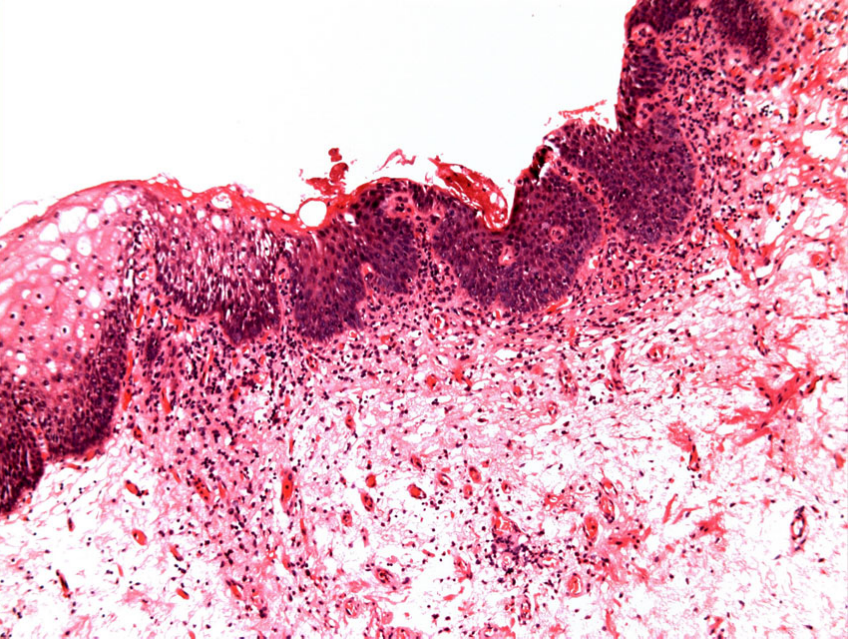
**Histology of bladder biopsy (18 June 1999) revealed inflamed bladder mucosa showing non-keratinising squamous metaplasia (top left surface) with probable focal keratinising metaplasia (top centre) (H&E stain)**.

X-ray of abdomen, taken on 17 June 1999, showed several vesical calculi. (Figure 3) On 18 June 1999, suprapubic cystolithotomy was performed. Bladder biopsy was taken. Temporary suprapubic cystostomy was done. Histology showed bladder mucosa covered entirely by squamous epithelium, which was predominantly of non-keratinising vaginal type, but there were small foci of keratinising squamous metaplasia also. (Figure 4) She was performing catheterisations through stoma in December 1999. On 24 March 2000, she was unable to insert a catheter. Emergency suprapubic cystostomy was performed. A 12 French catheter was inserted. Loopogram was done on 30 March 2000, which showed complete obstruction of stoma. The suprapubic catheter was getting blocked. She was prescribed ranitidine 150 milligrams, twice daily to decrease mucus production by intestinal segment. On 05 April 2000, suprapubic catheter was not draining. This patient was in agony. She tried to catheterise through Benchekroun stoma. But catheterisation was not possible. There was bleeding. After several attempts, the catheter was unblocked. On 07 April 2000, endoscopy was attempted through Benchekroun stoma. It was not possible to insert ureterorenoscope beyond two inches. There were mucosal tears. The track was completely blocked. Suprapubic cystostomy tract was dilated with filiform bougies. A 24 French dilator was successfully threaded through. Then a 20 French Foley catheter was inserted through suprapubic cystostomy tract. Conduitogram was performed in July 2000. A week later it got infected and there was cellulitis. Aspiration was attempted; no pus could be aspirated. Antibiotics were prescribed for ten days. On 04 August 2000, it was apparent that a fistula had developed from Benchekroun stoma to the skin.

**Figure 5:**
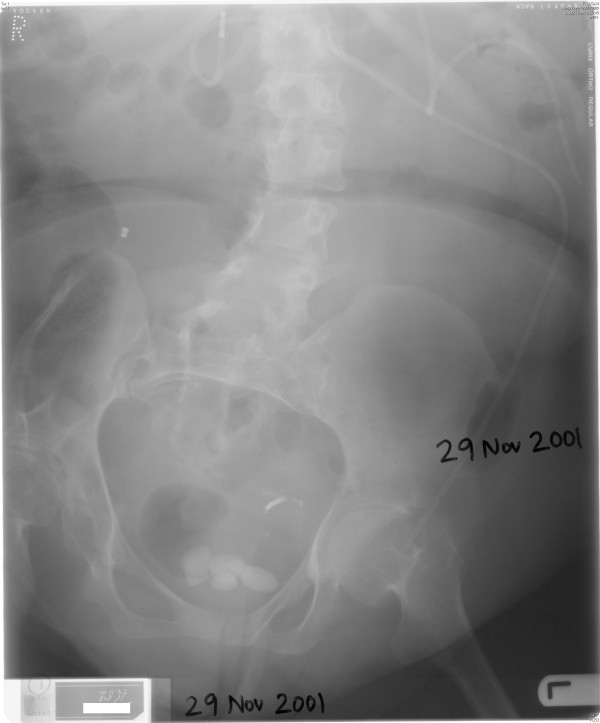
**X-ray of abdomen, taken on 29 November 2001, showed several vesical calculi**.

**Figure 6:**
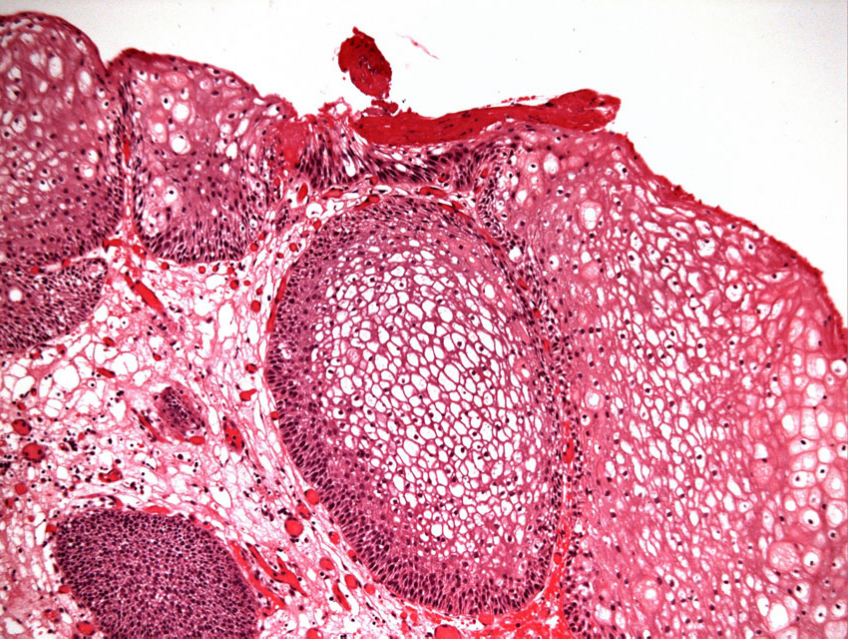
**Histology of bladder mucosal biopsy (30 November 2001) showed extensive non-keratinising squamous metaplasia, including in von Brunn's nests, with probable focal keratinising metaplasia (top centre) (H&E stain)**.

X-ray of abdomen, taken on 29 November 2001, showed several vesical calculi. (Figure 5) On 30 November 2001, suprapubic cystolithotomy was performed. A vertical incision was made from the lower border of suprapubic track. The opening in bladder was enlarged. All stones were removed. A 22 French catheter was kept for suprapubic drainage. A biopsy was taken from bladder mucosa. Histology showed vaginal type metaplastic squamous epithelium. However, in one area, there was probable surface keratinisation. (Figure 6) This patient developed dehiscence of wound and re-suturing was performed on 06 December 2001. This patient was experiencing leak of urine around suprapubic catheter. Urine leak was affecting patient's family as well.

Video urodynamics was attempted on 16 November 2004. Pressure-recording catheter was inserted alongside suprapubic catheter. Attempts at monitoring pressures were unsuccessful. The procedure was abandoned, as there was extravasation of contrast.

**Figure 7:**
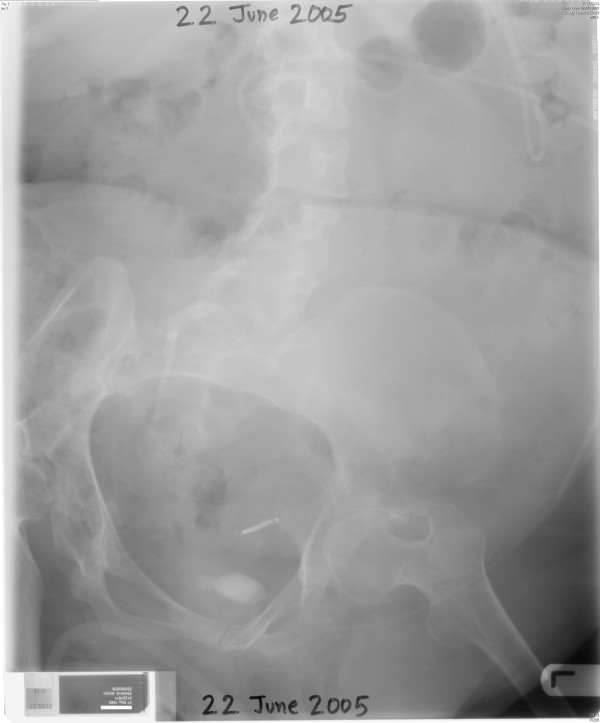
**X-ray of abdomen, taken on 22 June 2005, showed vesical calculi**.

In March 2005, this patient developed pain in abdomen. X-ray of abdomen, taken on 04 March 2005, showed a large opaque shadow in pelvis, probably a vesical calculus. X-ray of abdomen, taken on 22 June 2005, showed radio opaque shadows in the region of urinary bladder. (Figure 7) On 24 June 2005, suprapubic catheter was removed and a cystoscope was introduced in to the bladder. Then electrohydraulic lithotripsy was performed.

**Figure 8:**
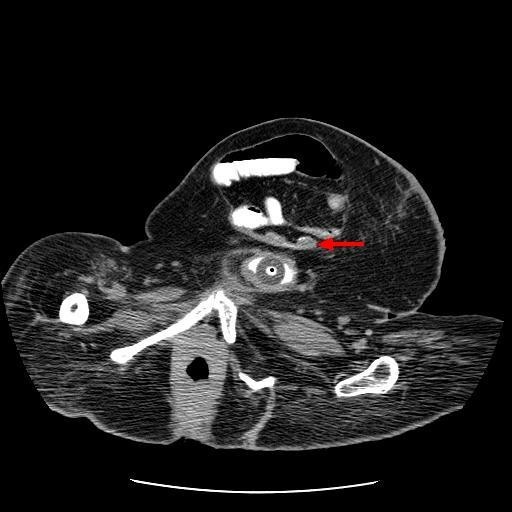
**Axial computed tomography of abdomen, performed on 19 June 2007, revealed midline, lower abdominal wall hernia, whose neck measured 45 millimetres wide**. Within the hernia, were several loops of small bowel and vesicostomy. (arrow).

**Figure 9:**
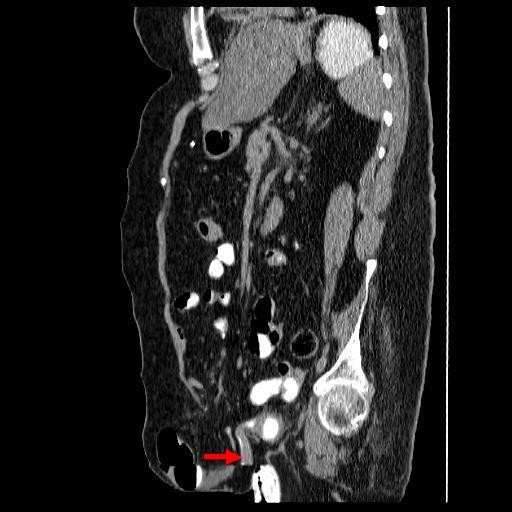
**Oblique sagittal reformat of computed tomography of abdomen, performed on 19 June 2007 revealed herniation of vesicostomy**. (arrow).

In 2007, this patient was concerned about the increasing swelling in lower abdomen. Clinical impression was large incisional hernia. Computed tomography of abdomen was performed on 19 June 2007. CT revealed midline, lower abdominal wall hernia, whose neck measured 45 millimetres wide. Within the hernia, were several loops of small bowel; one loop of bowel was dilated moderately. (Figure 8) The hernia also contained the ileal cystoplasty. (Figure 9) There was distension of gallbladder. A faintly opaque calculus was seen impacted in the neck of gallbladder. The large hernia was uncomfortable and was tender on coughing but did not cause obstructive bowel symptoms. Closure of hernial defect with prosthetic material was considered. But this patient might require ventilatory support following reduction of herniated bowel into the peritoneal cavity. Further, this patient would require alternative way of urinary diversion because the current location of suprapubic catheter would almost lead to infection of prosthetic material used in reconstruction of the anterior abdominal wall. The pros and cons of elective surgical repair of abdominal hernia were discussed with the patient and husband. The family reached informed decision of not proceeding with surgery.

## Discussion

Information regarding delayed complications of a surgical procedure is very important especially from patient's viewpoint. In spinal cord injury patients, urethral sphincterotomy is often portrayed as a successful procedure for treatment of detrusor-sphincter dyssynergia. But review of 84 patients undergoing external sphincterotomy at a large tertiary referral spinal injuries centre revealed that duration of successful outcome was only 81 months for primary sphincterotomies [2]. A second procedure was required in 30 patients and mean duration of success thereafter was 80 months. However, recurrent symptomatic episodes of urinary tract infection, recurrent detrusor-sphincter dyssynergia or upper tract dilatation eventually ensued in 57 of 84 patients (68%).

While recommending a surgical procedure to a spinal cord injury patient, physicians should provide results of long-term follow-up in order to assess benefits of proposed surgical procedure. Ileal conduit urinary diversion is recommended to spinal cord injury patients, who develop severe vesicourethral dysfunction. But follow-up of sixteen tetraplegic subjects, who underwent ileal conduit urinary diversion, revealed that five patients suffered from repeated renal or ureteral stone, and eight patients suffered from empyema of the bladder. Kato and associates from Department of Urology, Shinshu University School of Medicine in Matsumoto, Japan [3] concluded that ileal-conduit formation should be *cautiously *considered as an option in the urinary management of tetraplegic patients.

Complications of Benchekroun continent vesicostomy are not uncommon. Mouriquand and Boddy from Great Ormond Street Hospital, London [4] described four patients, who developed complications related to devagination, over-distension of the hydraulic valve, and stoma of Benchekroun valve. These authors concluded that complications or failure of the Benchekroun valve and its variants (e.g. Guzman's technique) were common, and salvage procedures were often necessary to recreate an efficient continent conduit.

This case reiterates the importance of discussing possible delayed complications of any surgical intervention with patients. Knowledge regarding risks of a procedure will help patients to make an informed decision as to whether they should agree to undergo a surgical procedure. Our patient developed series of adverse events following Benchekroun continent vesicostomy, which affected seriously the quality of her life.

## Conclusion

This case is a poignant reminder to spinal cord physicians that novel surgical techniques should be viewed cautiously. Physicians should inform patients of potential complications of surgical procedures, as some complications might be irreversible and adversely affect quality of life.

## Patient's perspective

➢ After having my Mitrofanoff performed around 20 years ago, I was dry for a while. One day, it stopped working. In the middle of the night, I was rushed to spinal unit, Southport where the doctors had to do an urgent suprapubic cystostomy.

➢ The thing about Mitrofanoff I did not like was that I got bladder stones a lot. I had to get stones removed surgically several times.

➢ Suprapubic cystostomy works well most of the time. But I do get some leakage of urine around suprapubic site.

➢ I have an abdominal hernia. When I cough or sneeze, it hurts.

➢ The abdominal hernia and leak of urine cause problems in my marriage.

## Competing interests

The authors declare that they have no competing interests.

## Authors' contributions

SV developed the concept and wrote draft. BMS was the Consultant in charge of this patient. SV assisted all urological procedures. GS performed surgery for removal of stones from urinary bladder. PH reviewed medical images. PM reported bladder biopsies. All authors read and approved the final manuscript.

## Consent

Written informed consent was obtained from the patient for publication of this case report and accompanying images. A copy of the written consent is available for review by the Editor-in-Chief of this journal.
